# How does malaria in pregnancy impact malaria risk in infants?

**DOI:** 10.1186/s12916-018-1210-8

**Published:** 2018-11-20

**Authors:** Prasanna Jagannathan

**Affiliations:** Department of Medicine, Stanford University, Stanford, USA

**Keywords:** Malaria, innate immunity, malaria in pregnancy, cord blood, TLR stimulation

## Abstract

Malaria in pregnancy not only exerts profound negative consequences on the health of the mother and developing fetus, but may also alter the risk of malaria during infancy. Although mechanisms driving this altered risk remain unclear, in utero exposure to malaria antigens may impact the development of fetal and infant innate immunity. In an article in *BMC Medicine*, Natama et al. describe an ambitious analysis of basal and TLR-stimulated cord blood responses among a birth cohort in Burkina Faso. Basal levels of several cytokines, chemokines, and growth factors were shown to be significantly lower in cord blood with histopathologic evidence of placental malaria. Additionally, following TLR7/8 stimulation, samples obtained from infants of mothers with placental malaria were hyper-responsive compared to those without evidence of prenatal malaria exposure. Furthermore, several responses impacted by placental malaria were associated with differential malaria risk in infancy. Understanding how malaria in pregnancy shapes immune responses in infants will provide critical insight into the rational design of malaria control strategies during pregnancy, including intermittent preventative treatment in pregnancy and vaccines.

Please see related article: https://bmcmedicine.biomedcentral.com/articles/10.1186/s12916-018-1187-3

## Background

Malaria in pregnancy remains a significant problem across sub-Saharan Africa, exerting profound negative consequences on the health of the mother and developing fetus [1]. Additionally, there is increasing evidence that malaria exposure in utero may alter the risk of malaria and non-malarial febrile infections in infancy, with studies showing that offspring of women who experience malaria in pregnancy are at increased risk of malaria themselves [2–5]. However, it remains unclear whether these associations are a direct result of malaria in pregnancy or rather reflect shared environmental exposures between maternal–infant pairs.

The use of multiple definitions of malaria exposure during pregnancy complicates the evaluation of the impact of such exposure on infancy. Although *Plasmodium falciparum* infection in pregnancy can be detected in maternal peripheral blood, it is also sequestered in the placenta through binding to chondroitin sulfate A expressed on the placental syncytiotrophoblast via the parasite molecule VAR2CSA [6, 7]. Importantly, not all women with *P. falciparum* infection detected in peripheral blood have evidence of placental malaria [8], and these overlapping definitions of malaria exposure in pregnancy may exert differential impacts on the developing fetus.. Further, several strategies for diagnosing placental malaria can be utilized, including assessment of placental blood to detect parasites and histopathologic evaluation to detect parasites, hemozoin pigment, or both. Identification and classification of these pathologic findings as either active infection (detection of parasites with (acute) or without (chronic) hemozoin) versus past infection (detection of hemozoin pigment alone) has been associated with differential impacts on infant outcomes, including adverse birth outcomes such as preterm birth [6, 9–11].

Although there are several potential mechanisms by which maternal malaria may impact the risk of malaria in infancy, it is increasingly appreciated that malaria in pregnancy may directly impact the development of the fetal and infant immune system [12, 13]. However, the precise mechanisms by which malaria in pregnancy may impact the risk of malaria in infancy remain elusive. Furthermore, it remains unclear whether alterations in fetal and infant immunity induced by malaria in pregnancy are then causally responsible for alterations in malaria risk in infants.

## Impact of malaria in pregnancy on innate immune responsivity in infants

Several studies have investigated the effect of malaria in pregnancy on innate immune responses in the neonate, particularly focusing on the activation of antigen presenting cells following stimulation with toll like receptor (TLR) ligands [14–16]. These studies have tested the hypothesis that in utero malaria antigen exposure may drive abnormal antigen presenting cell activation, leading to parasite-specific tolerance and an increased risk of infection in infants. Stimulation with polyinosinic-polycytidylic acid (TLR3), LPS (TLR4), and/or CpG oligonucleotide type A (TLR9) has been associated with altered cytokine production in whole cord blood [16] or cord blood mononuclear cells [14, 15] isolated from infants born to mothers exposed to malaria in pregnancy. Furthermore, increased cord blood production of IL-10 after TLR3 or TLR7/8 (resiquimod) stimulation was associated with an increased risk of *P. falciparum* infection during infancy [16], suggesting clinical consequences of differential TLR signaling at birth. However, these studies were limited by the panel of cytokines tested, as well as by the varying (and non-specific) definitions of malaria exposure in pregnancy.

Natama et al. [17] undertook an ambitious analysis of cord blood innate cell responsivity to TLR stimulation among a well-characterized birth cohort in a highly malaria endemic setting in Burkina Faso, posing two overarching questions. Firstly, what impact do different manifestations of malaria in pregnancy have on a broad panel of cytokines, chemokines, and growth factors measured at birth, both at baseline and following TLR-stimulation? Secondly, is basal or TLR-stimulated cytokine production at birth associated with protection from malaria in infancy? The study involved cord blood obtained from 313 maternal–infant pairs enrolled in a clinical trial in Burkina Faso assessing novel interventions to prevent malaria in pregnancy [18]. In this trial, pregnant women were enrolled and followed by both active and passive surveillance for malaria infection during pregnancy; at delivery, placental tissue was examined for histopathologic evidence of placental malaria as defined above. Infants born to these mothers were followed through 1 year of age. Natama et al. [17] assayed a panel of 30 cytokines, chemokines, and growth factors in whole cord blood supernatants by Luminex following stimulation with TLR3, 7/8, and 9 agonists, or unstimulated controls. The authors first looked for associations between malaria exposure in pregnancy and these immune features, and then evaluated whether basal or TLR-stimulated immune profiles at birth were associated with differential malaria risk in the first year of life. Basal levels of several immune features, including cytokines (e.g., IFN-α, IL-1β, IL-1RA, TNF, IFN-γ, IL-10), chemokines (e.g., MIP-1α, Rantes), and growth factors (e.g., G-CSF, GM-CSF, FGF), were found to be significantly lower in samples with evidence of malaria in pregnancy than in those that were unexposed. However, cord blood samples obtained from infants with evidence of ‘past’ placental malaria showed increased responsivity to TLR7/8 stimulation.

One potential explanation for these results is the possibility of differential admixture of cells in cord blood from infants exposed to malaria in utero, though cord blood cellular populations were not measured in this study. Indeed, malaria in pregnancy has been associated with increased myeloid dendritic cells in cord blood [19, 20] and malaria pigment in the placenta has also been associated with ‘partial maturation’ of cord blood myeloid and plasmacytoid dendritic cells [15]. However, an alternative explanation is that malaria exposure in pregnancy may alter innate cell responsivity, including the possibility that malaria exposure may induce ‘trained’ innate immunity, as has recently been suggested [21], though this remains to be determined.

Importantly, the authors found that several immunologic features impacted by placental malaria exposure were also associated with differential malaria risk in infancy. For example, higher concentrations of GM-CSF and eotaxin following TLR7/8 stimulation, of IL-1β following TLR9 stimulation, and of IL-7 following IL-3 stimulation, were associated with an increased hazard of malaria in the first year of life. In contrast, a higher concentration of IP-10 following TLR3 or TLR9 stimulation was associated with a lower hazard of malaria. Taken together, these data suggest that placental malaria may influence cord blood responsivity, and that these alterations may impact the subsequent risk of malaria early in life.

## Conclusion

Malaria during pregnancy may lead to significant and long-lasting effects on the infant, including a predisposition to a greater risk of malaria in early life. By finding that placental malaria may impact innate immune responsivity in infants, and that these alterations may be associated with differential malaria risk in infants, Natama et al. [17] suggest a potential mechanism for this epidemiologic association. Future studies will need to evaluate whether (and how) malaria in pregnancy may perturb innate cellular populations, including whether placental malaria may drive intrinsic changes within these cells. Furthermore, mechanistic studies should attempt to determine whether these immunologic correlates are causally responsible for the associations observed. An improved understanding of how malaria in pregnancy shapes immune responses in infants may provide important insights into the rational design and development of malaria control strategies in pregnancy.
